# A deep neural network and AIS-integrated method for ship trajectory prediction and yaw warning in cross-river power transmission line protection

**DOI:** 10.1371/journal.pone.0350221

**Published:** 2026-06-09

**Authors:** Yang Rui, Liang Zhaofeng, Liang Xunyun, Liu Zehuai

**Affiliations:** DongGuan Power Supply Bureau of Guangdong Power Grid Co., Ltd., DongGuan, Guangdong, China; Beijing Institute of Technology, CHINA

## Abstract

Ship trajectory prediction plays a crucial role in ensuring the safety of inland waterway transportation and enabling intelligent scheduling. To address the limitations of traditional models in capturing long-term dependencies and extracting salient features, this study proposes a novel prediction model—TDV-TTCN-BiGRU—integrating a Temporal-Dependent Variable (TDV) attention mechanism with an improved TCN-BiGRU architecture. The model employs a hierarchical Temporal Convolutional Network (TTCN) to extract multi-scale temporal features in parallel, incorporates the TDV attention mechanism to adaptively adjust the weights of speed and heading features, and uses BiGRU to model bidirectional dependencies, thereby enhancing prediction accuracy and stability. Experiments based on real AIS data include both comparative and ablation studies. Results show that the proposed TCN-BiGRU outperforms CNN, BiGRU, CNN-BiLSTM, and CNN-BiGRU models, achieving the lowest prediction errors. Compared to CNN-BiGRU, the TDV-TTCN-BiGRU model reduces MSE of predicted longitude and latitude by 14.47% and 18.83%, respectively; MAE by 22.41% and 21.25%; and ADE by 21.46%, with trajectory plots showing closer alignment with actual vessel tracks. Furthermore, to address the risk of vessels deviating from navigable channels, a multi-level yaw warning mechanism is developed and validated in typical cross-river scenarios. The system achieves a warning accuracy of over 96%, significantly improving the responsiveness to unexpected yaw behavior. The proposed method provides technical support for intelligent ship navigation, maritime safety management, and the protection of overhead transmission lines.

## Introduction

With the rapid development of the shipping industry, the number of vessels navigating inland waterways has surged. However, due to factors such as violations by heavy vessel operators, negligence, and insufficient training in electrical safety, incidents involving ships colliding with overhead power transmission lines that span across waterways have occurred with increasing frequency. These accidents not only pose serious threats to vessel safety and human life but also jeopardize the stability of power supply systems. As a result, the development of advanced and reliable ship trajectory prediction and yaw warning methods has become a research hotspot in inland waterway traffic safety. Such technologies are crucial for enhancing navigation safety and protecting cross-river transmission lines from external mechanical damage.

To date, substantial research has been conducted on ship trajectory prediction by scholars both domestically and internationally. Traditional methods are mainly categorized into kinematics-based models and machine learning-based approaches. Kinematic models often suffer from significant errors when dealing with nonlinear dynamic behaviors and heavily depend on state transition probabilities, resulting in limited adaptability and accuracy in complex navigation environments. Machine learning methods, on the other hand, struggle to fully exploit the temporal features of AIS (Automatic Identification System) data and are prone to parameter tuning challenges and local optima, which compromise prediction performance.

In recent years, with the advancement of deep learning, neural network-based models have gradually become the mainstream approach for trajectory prediction. Ma et al. [1] proposed a multi-trajectory prediction model based on RNN-LSTM. Xue et al. [2] developed a hierarchical architecture integrating GRU and Transformer, significantly improving mid- and long-term prediction capabilities. Wang [3] combined CNN and LSTM for AIS trajectory modeling, enhancing spatial and temporal feature extraction. Zhao et al. [4] introduced a feature attention mechanism into the RNN-BiLSTM framework, notably improving prediction accuracy, especially in curved navigation scenarios. GRU, known for its fewer parameters and faster training speed, has also been widely used in trajectory modeling. You et al. [5] utilized GRU to encode spatiotemporal AIS sequences, mitigating gradient vanishing and improving model performance. Yu et al. [6] further integrated ARIMA and BiGRU with attention mechanisms to model ship trajectories. Moreover, Temporal Convolutional Networks (TCN) have drawn attention for combining the parallelism of convolutional operations with the capability to model long-term dependencies. Guo [7] proposed an attention-based model integrating TCN and Bi-LSTM to effectively extract past and future trajectory states. Dong et al. [8] developed a CNN-MTABiGRU model incorporating temporal attention, which demonstrated significant error reduction in prediction tasks.

Despite the notable progress achieved by deep learning-based approaches in ship trajectory prediction, challenges remain in terms of limited feature extraction capability and insufficient generalization [9]. Especially in complex inland waterway environments, existing models often fail to fully capture long-term dependencies in temporal sequences, resulting in unsatisfactory prediction accuracy for practical applications [10]. Thus, designing models with enhanced temporal perception and feature extraction abilities remains a pressing research challenge.

To address these issues, this paper proposes a novel ship trajectory prediction and yaw warning method that integrates a Temporal-Dependent Variable (TDV) attention mechanism with an improved TCN-BiGRU framework. A hierarchical TCN (TTCN) is introduced to overcome the limitations of conventional TCN in long-sequence modeling, leveraging multi-layer parallel dilated convolutions to enhance trajectory feature extraction and improve the model’s capability to handle long-term dependencies. Meanwhile, the TDV attention mechanism adaptively adjusts the feature weights of navigational directions (i.e., heading and speed), optimizing prediction performance under varying vessel motion states. BiGRU is then incorporated to further model the temporal dependencies in trajectory data, enhancing the overall prediction accuracy and stability. Experimental results verify the accuracy and robustness of the proposed model. Furthermore, in the tested river segments, the model successfully issues safety prompts and yaw warnings to passing vessels, thereby improving the management and emergency response capacity of cross-river transmission lines. The proposed method significantly improves the accuracy, reliability, and robustness of trajectory prediction in inland waterway environments, offering technical support for intelligent waterway management and the protection of overhead power transmission lines, with promising application and promotion value.

## Model architecture

### Temporal convolutional network

**Fig 1:**
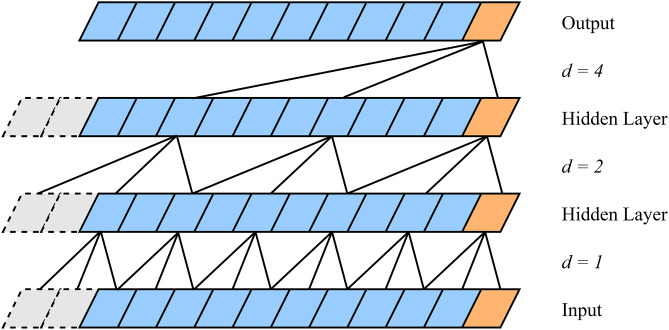
Structure of dilated causal convolution.

The Temporal Convolutional Network (TCN) is an architecture that integrates the core concepts of Convolutional Neural Networks (CNNs) and is specifically designed to handle time series data. It enables more efficient parallel computation and offers flexible receptive fields, thereby providing advantages in both training and inference stages. TCN employs a dilated causal convolution structure that ensures temporal causality while achieving a large receptive field with fewer network layers, which is critical for modeling long-term dependencies [11]. The structure of a dilated causal convolution is illustrated in Fig 1.

As shown in Fig 1, input data enters the network from the lower layers, where each data point is processed through convolutional layers. The dilation factor determines the step size between input points and is commonly set to increase exponentially, such as 1, 2, 4, etc. By progressively increasing across layers, TCNs can achieve a large receptive field with relatively few layers, enabling them to capture long-range temporal dependencies. The total receptive field after L stacked layers can be expressed as:


$$\begin{array}{c} R_{L}=1+(k-1)\sum_{l=0}^{L-1}d_{l}=1+(k-1)({\text{2}}^{L}-1) \end{array}$$ (1)

where k denotes the kernel size, and $d_{l}$ is the dilation factor at the l -th layer. With exponential growth in d, the convolutional kernel can effectively capture information across a broad temporal span. The output layer aggregates the convolutional features from the previous layers and produces the final output of the network. This structure combines the strengths of causal and dilated convolutions, enabling TCNs to adaptively expand their receptive fields layer by layer and exhibit strong performance in time series modeling and forecasting tasks.

**Fig 2:**
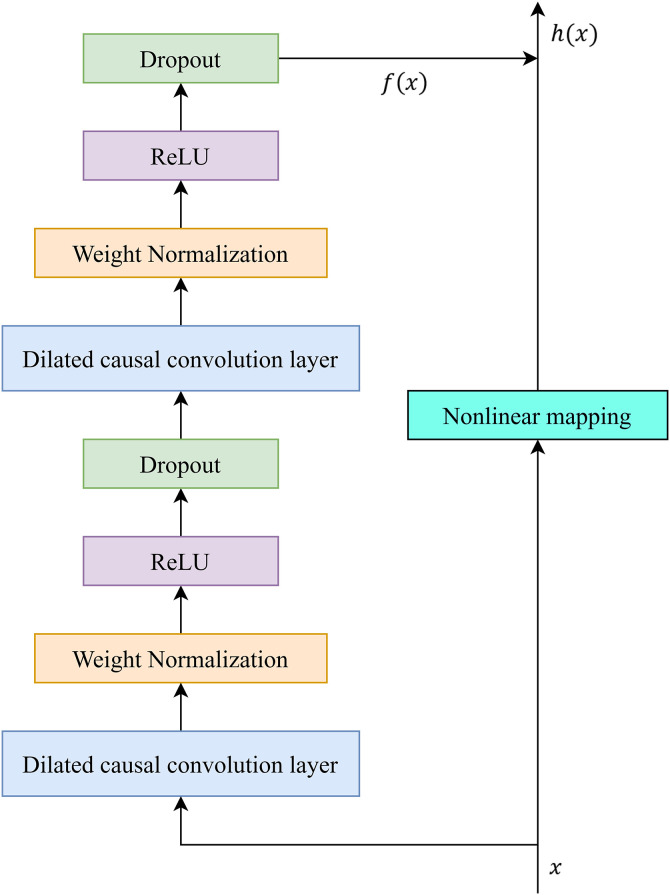
Residual block in traditional TCN.

To address the vanishing gradient problem as networks deepen, residual connections, as proposed by He et al. [12], are incorporated to improve training efficiency and model performance. Each residual block in the TCN comprises four components: dilated causal convolution, weight normalization, an activation function, and regularization. The structure of a standard TCN residual block is shown in Fig 2.

In a residual connection, the output from one layer is directly added to the output of a subsequent layer through a “skip connection.” Specifically, if the input is x and the output of the transformation is f(x), the residual output becomes x+f(x). This residual formulation can be mathematically expressed as Equation (2). Residual connections facilitate stable gradient flow throughout the network and enhance training efficiency.


$$\begin{array}{c} h\left(x\right)=f\left(x\right)+x \end{array}$$ (2)

**Fig 3:**
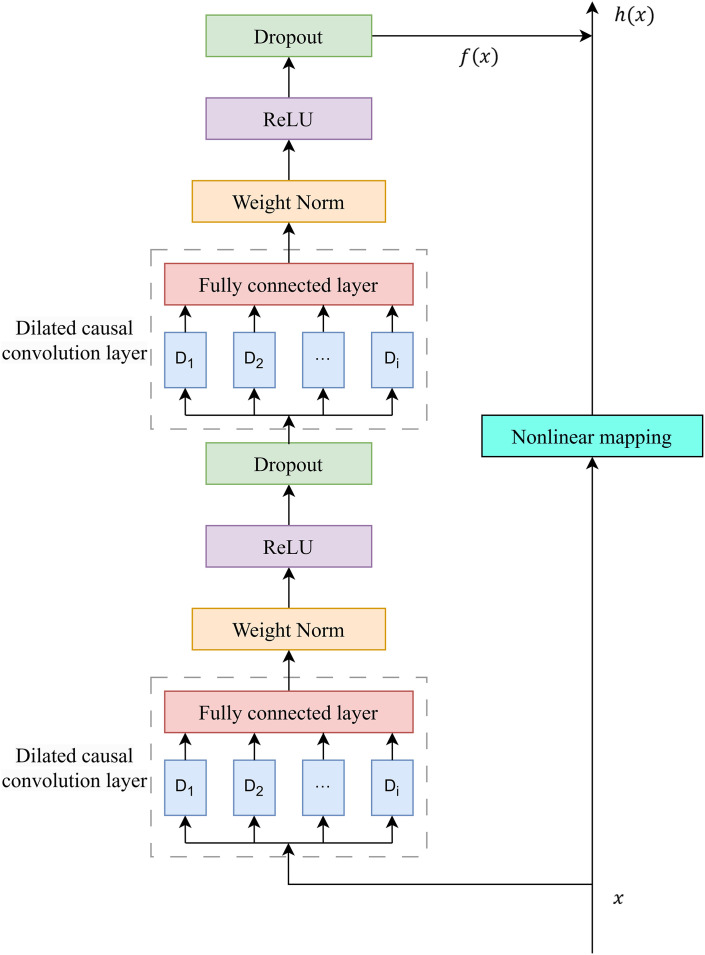
Architecture of the proposed TTCN model.

In traditional TCNs, each layer employs only a single dilated causal convolution with a fixed dilation factor to expand the receptive field. This design suffers from a critical limitation: a single temporal scale is insufficient to capture complex temporal dependencies effectively. To overcome this, we propose a Tiered Temporal Convolutional Network (TTCN), which structurally expands the single-layer convolution into a multi-branch parallel design. Specifically, each residual unit contains multiple parallel dilated causal convolution layers with varying dilation factors. This multi-scale feature extraction mechanism enables the model to simultaneously capture both short-term and long-term temporal patterns. The outputs from these parallel branches are then fused via a fully connected layer to ensure that essential information is fully utilized. The improved residual unit structure is shown in Fig 3.

In this figure, $D_{\text{1}},D_{\text{2}},\ldots ,D_{i}$ represent dilated causal convolution layers with different dilation factors. The number of parallel branches i must be chosen carefully, balancing model complexity, temporal characteristics of the data, and computational resource constraints. Generally, a smaller i is suitable for modeling short-term dependencies with reduced computational cost and faster training, whereas a larger i is more appropriate for long-sequence forecasting at the expense of higher computational demands. In this study, we set the number of parallel dilated convolution branches to i=3, which achieves low prediction error while maintaining computational efficiency.

In summary, the improved TTCN architecture—through its parallel dilated convolution layers, multi-scale feature fusion, and optimized residual blocks—enables more accurate modeling of ship trajectory data. Compared with traditional TCNs, this structure allows parallel feature extraction across different temporal scales, enhancing the model’s ability to capture multi-scale temporal dependencies and long-term trajectory trends. This serves as a solid foundation for the subsequent integration of attention mechanisms to further optimize the model.

### TDV attention mechanism

The attention mechanism was initially introduced for machine translation tasks and has since been widely applied in areas such as speech recognition and image processing [13]. Its core idea lies in dynamically assigning weights to each time step in the input sequence based on their importance, thereby extracting more representative features [14].

In the context of ship trajectory prediction, although traditional attention mechanisms can capture sequence features, they fall short in modeling directional changes and motion trends within trajectories. To address this limitation, we propose the TDV (Trajectory Direction Vector) Attention Mechanism. The central idea of TDV is to integrate a ship’s Speed Over Ground (SOG) and Course Over Ground (COG) to enhance the model’s ability to capture motion dynamics. The attention mechanism is then used to adaptively adjust the feature weights of the trajectory, improving both prediction accuracy and stability.

**Fig 4:**
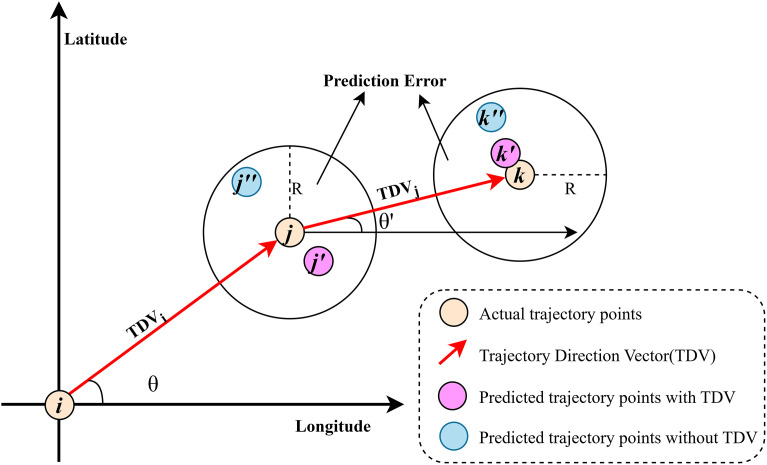
Schematic of predicted weight distribution using TDV attention mechanism.

This mechanism combines traditional attention with trajectory direction information. During prediction, it dynamically adjusts the weights of SOG and COG, thereby improving the model’s adaptability to different navigation phases (e.g., acceleration, deceleration, turning). A schematic diagram illustrating the predicted weight distribution under the TDV attention mechanism is shown in Fig 4.

**Fig 5:**
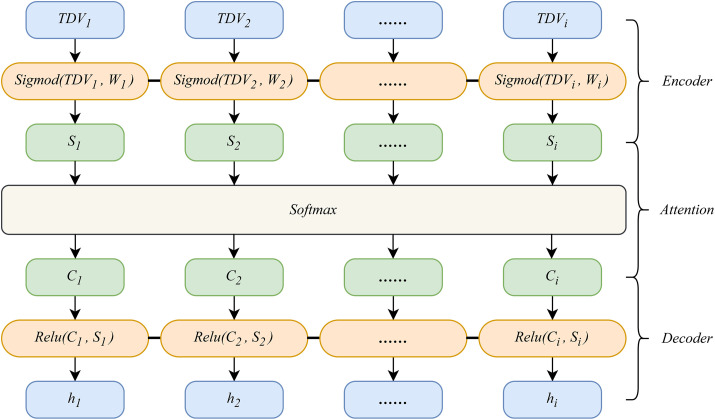
Architecture of the TDV attention mechanism.

In this figure, i represents a known trajectory point, for which the latitude, longitude, sog, and cog are known. θ denotes the navigation direction from point i to j. $TDV_{i}$ represents the trajectory direction vector, which can be interpreted as the product of sog and the time interval, i.e., the sailing distance. R indicates the prediction error range; a smaller R implies a better prediction. $\begin{array}{c} j^{\prime } \end{array}$ is the predicted trajectory point generated by the deep learning model enhanced with the TDV attention mechanism, whereas $\begin{array}{c} j^{\prime \prime } \end{array}$ is predicted by a traditional deep learning model without TDV. The latter exhibits significantly higher error. As training iterations progress, the TDV component adaptively adjusts the weight distribution of $\begin{array}{c} j^{\prime } \end{array}$, addressing random prediction deviations and enhancing accuracy. The structure of the TDV attention mechanism is illustrated in Fig 5, which consists of three main components: Encoder, Attention Mechanism, and Decoder.

As shown above, in the encoder part, the input trajectory data is processed to extract features, and the trajectory direction information is encoded into hidden state vectors that serve as inputs to the attention mechanism. The trajectory direction vector **TDV** is defined as:


$$\begin{array}{c} 𝐓𝐃{𝐕}_{𝐢}=sog_{i}\cdot \Delta t_{i}\cdot cos\left(\frac{cog_{i}}{180^{\circ }}\right) \end{array}$$ (3)

where $sog_{i}$ is the speed over ground at time step i, $cog_{i}$ is the course over ground at that moment, and $\Delta t_{i}$ is the time interval. These values are then input into the encoder to produce hidden states, calculated as follows:


$$\begin{array}{c} {𝐒}_{𝐢}=sigmod({𝐖}_{𝐞}𝐓𝐃{𝐕}_{i}+{𝐔}_{𝐞}{𝐒}_{𝐢-1}) \end{array}$$ (4)

where ${𝐖}_{𝐞}$ and ${𝐔}_{𝐞}$ are the weight matrices for the input layer and hidden state **S**, respectively, and sigmoid is a nonlinear activation function. The encoder’s function is to extract trajectory features and represent them in a high-dimensional space, which are then passed to the attention module for adaptive weight adjustment during prediction.

In the attention mechanism, the model evaluates the importance of different trajectory features based on the encoder’s output and assigns different weights. For each trajectory point ${𝐒}_{𝐢}$ and decoder hidden state ${𝐡}_{𝐢}$, a similarity score is computed using trainable parameters ${𝐖}_{𝐚}$ and ${𝐔}_{𝐚}$:


$$\begin{array}{c} e_{i}=tanh({𝐖}_{𝐚}{𝐒}_{𝐢}+{𝐔}_{𝐚}{𝐡}_{𝐢}) \end{array}$$ (5)

These similarity scores are normalized using the Softmax function to derive attention weights:


$$\begin{array}{c} {𝐚}_{𝐢}=\frac{exp\left(e_{i}\right)}{\sum_{j=1}^{n}exp\left(e_{j}\right)} \end{array}$$ (6)

Finally, the weighted sum of all trajectory points is computed to obtain the context vector:


$$\begin{array}{c} {𝐂}_{𝐢}=\sum_{j=1}^{n}{𝐚}_{𝐣}{𝐒}_{𝐣} \end{array}$$ (7)

The context vector ${𝐂}_{𝐢}$ encapsulates the most relevant historical trajectory information needed for predicting the current ship location and is passed to the decoder for the next step.

In the decoder part, the context vector ${𝐂}_{𝐢}$ is combined with the previous decoder hidden state $h_{i-1}$ to predict the current ship trajectory point. The computation is given by:


$$\begin{array}{c} {𝐡}_{𝐢}=ReLU({𝐖}_{1}{𝐡}_{𝐢-1}+{𝐖}_{2}{𝐒}_{𝐢-1}+{𝐔}_{𝐝}{𝐂}_{𝐢}+b) \end{array}$$ (8)

Where ${𝐖}_{\text{1}}$ is the weight matrix of 𝐡, ${𝐖}_{2}$ is the weight matrix of 𝐒, and ReLU is the activation function. The decoder output ${𝐡}_{𝐢}$ is then projected into the trajectory prediction space via a fully connected layer to generate the predicted value for the next time step.

The TDV attention mechanism dynamically adjusts feature weights by incorporating trajectory direction vectors formed from SOG and COG. It demonstrates heightened sensitivity during significant maneuvering events such as acceleration, deceleration, and turning, while automatically reducing the influence of trajectory features during stable cruising to minimize noise. This mechanism compensates for the temporal lag often encountered in traditional attention methods, significantly improving both the accuracy and stability of trajectory prediction.

Compared with existing studies, the TDV attention mechanism proposed in this paper exhibits distinct differences and extensions in both design and application. First, previous works (e.g., Chen [15]) have explored TDV-based approaches to adaptively adjust the weights of heading and speed features across different navigation stages in order to enhance temporal modeling capability. However, most of these methods directly operate on the input layer or hidden states of neural networks, lacking integration with multi-scale convolutional features. In this study, the TDV attention mechanism is embedded into the improved TTCN-BiGRU framework, enabling it to dynamically select and weight critical heading and speed information at the multi-scale convolutional feature layer, thereby achieving cross-scale temporal feature filtering. Second, beyond verifying the effectiveness of TDV attention in trajectory prediction, this research further couples it with the logic of yaw deviation warning, forming a closed-loop mechanism of “prediction–evaluation–warning.”

### Bidirectional gated recurrent unit

**Fig 6:**
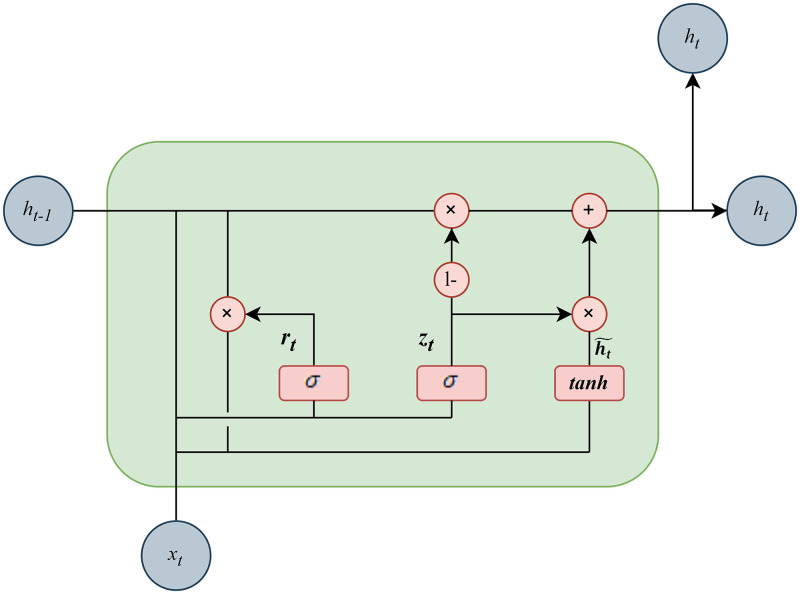
GRU model architecture.

The Gated Recurrent Unit (GRU) is an improved variant of the Recurrent Neural Network (RNN). By incorporating gating mechanisms, GRU effectively mitigates the vanishing gradient problem and enhances training efficiency while preserving long-term dependency information [16]. The structure of the GRU model is illustrated in Fig 6.

The GRU consists of two core components: the reset gate and the update gate. The process of state update is as follows:

The reset gate controls the influence of the previous hidden state on the current input:


$$\begin{array}{c} r_{t}=\sigma ({𝐖}_{𝐫}\cdot x_{t}+{𝐔}_{𝐫}\cdot h_{t-1}+b_{r}) \end{array}$$ (9)

The update gate determines how much historical information is retained in the current state:


$$\begin{array}{c} {\text{z}}_{t}=\sigma ({𝐖}_{z}\cdot x_{t}+{𝐔}_{z}\cdot h_{t-1}+b_{z}) \end{array}$$ (10)

The candidate hidden state is updated by combining the current input with the reset-modulated historical state [17]:


$$\begin{array}{c} \tilde{h_{t}}=tanh(W_{h}\cdot x_{t}+U_{t}(r_{t}⊙h_{t-\text{1}})+b_{h}) \end{array}$$ (11)

The final hidden state is computed by merging historical and new information under the control of the update gate:


$$\begin{array}{c} h_{t}=z_{t}⊙h_{t-\text{1}}+(\text{1}-z_{t})⊙\tilde{h_{t}} \end{array}$$ (12)

In these equations, σ() represents the Sigmoid activation function, ⊙ denotes the Hadamard (element-wise) product, $x_{t}$ is the current input, $\tilde{h_{t}}$ is the candidate state, $h_{t}$ is the hidden state, and **W**, **U** are trainable weight matrices with as the bias term.

Compared with standard RNNs, GRU is more effective in learning long-term dependencies and avoids the vanishing gradient issue. Compared with Long Short-Term Memory (LSTM) networks, GRU has fewer parameters, leading to shorter training times and reduced risk of overfitting, thereby improving computational efficiency [18].

However, the standard GRU processes sequence information only in the forward direction, making it difficult to capture full contextual dependencies. To enhance the temporal modeling capability, we introduce a Bidirectional GRU (BiGRU) structure, which allows the model to simultaneously integrate both forward and backward information, thereby enhancing its ability to perceive global trajectory features.

**Fig 7:**
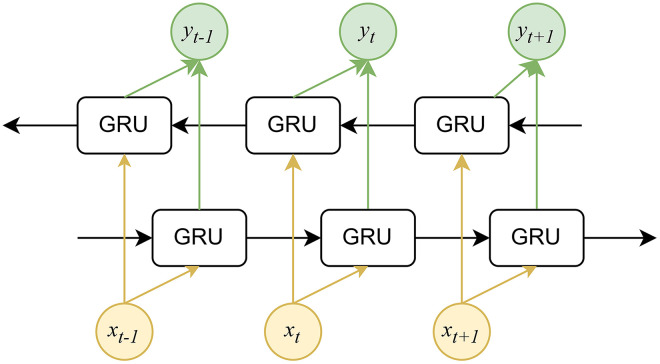
BiGRU model architecture.

The BiGRU consists of a forward GRU and a backward GRU, enabling the model to consider both past and future contexts and significantly improve time-series modeling. The structure of the BiGRU model is shown in Fig 7.

Compared to the unidirectional GRU, BiGRU substantially enhances the model’s ability to capture complex temporal patterns without significantly increasing computational complexity [19]. By integrating both forward and backward information flows, BiGRU achieves superior performance in many practical applications, particularly in tasks that require modeling long-term dependencies, outperforming traditional RNNs and unidirectional GRUs.

### TTCN-TDV-BiGRU trajectory prediction model

Although TCN possesses multi-scale convolutional receptive fields that can capture both local and mid-range dependencies of trajectories, the convolutional kernel weights remain fixed over time, limiting its adaptive memory capability in handling abrupt state changes (e.g., sharp turns or deceleration). The introduction of BiGRU addresses this issue by leveraging gating mechanisms to dynamically update hidden states, thereby enabling the model to adaptively retain or discard historical information within long sequences. While TCN is responsible for parallel extraction of multi-scale temporal features, BiGRU performs re-gating and integration along the temporal dimension. Together, they complement each other and significantly enhance the stability and generalization capability of trajectory prediction.

**Fig 8:**
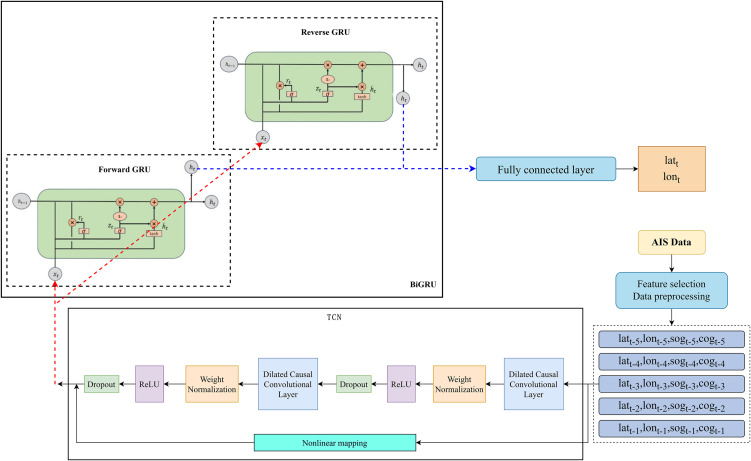
TCN-BiGRU ship trajectory prediction model.

To leverage the respective strengths of Temporal Convolutional Networks (TCN) and Bidirectional Gated Recurrent Units (BiGRU), this study constructs a ship trajectory prediction model based on the TCN-BiGRU architecture. The TCN is responsible for extracting both local and global temporal features from the trajectory data, while the BiGRU further learns the dynamic evolution patterns to optimize trajectory prediction. The structure of the TCN-BiGRU model is shown in Fig 8.

The detailed execution process of the TCN-BiGRU ship trajectory prediction model is as follows:

Step 1: Data Input and Preprocessing.

The raw AIS data is first filtered and preprocessed. The model takes the previous five time steps of longitude, latitude, Speed Over Ground (SOG), and Course Over Ground (COG) as input features for predicting the next time step.

Step 2: Temporal Feature Extraction via TCN.

The TCN utilizes causal convolutions to maintain the temporal order of the sequence, and dilated convolutions to enlarge the receptive field for capturing long-term dependencies. Weight normalization stabilizes the data distribution and accelerates model convergence. The ReLU activation function enhances nonlinear representation capability, and Dropout is employed to prevent overfitting and improve generalization. TCN extracts high-dimensional temporal features and passes them to the next stage.

Step 3: Sequence Modeling via BiGRU.

The BiGRU consists of a forward GRU and a backward GRU, enabling the model to learn temporal patterns in both directions and better capture the dynamic evolution of ship trajectories, thereby enhancing prediction accuracy.

Step 4: Trajectory Prediction via Fully Connected Layer.

The high-dimensional output features of the BiGRU are passed through a fully connected layer for nonlinear transformation, and finally, the predicted longitude and latitude at time t are obtained as output.

However, the standard TCN-BiGRU model lacks sufficient capacity to capture rapid changes in ship speed and direction, making it less effective in modeling directional dynamics embedded in the trajectory data. To further improve the prediction accuracy and robustness, this study proposes an enhanced model—TDV-TTCN-BiGRU—which integrates a TDV attention mechanism with an improved TCN-BiGRU structure.

**Fig 9:**
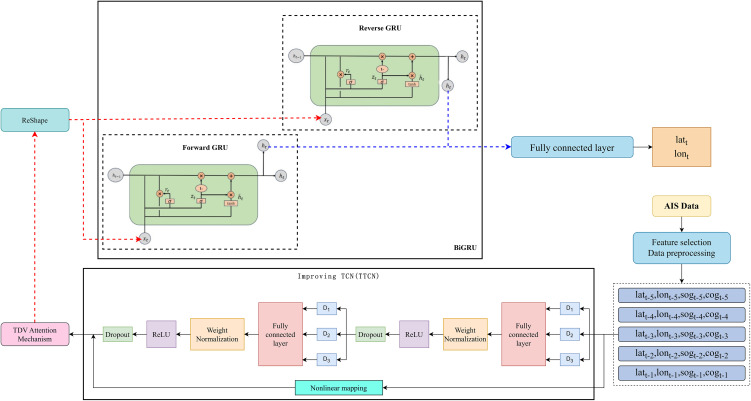
TDV-TTCN-BiGRU ship trajectory prediction model.

The core idea of the proposed model is to use an enhanced TCN (TTCN) to extract local temporal features of the ship trajectory, employ a Trajectory Direction Vector (TDV) attention mechanism to capture directional features, and leverage BiGRU for long-term dependency modeling, thereby obtaining a complete representation of trajectory dynamics and improving both accuracy and stability. The model architecture is illustrated in Fig 9.

The detailed execution process of the TDV-TTCN-BiGRU model is as follows:

Step 1: Data Input and Preprocessing.

As in the baseline model, the AIS data is filtered and preprocessed. The previous five time steps of longitude, latitude, SOG, and COG are selected as input features.

Step 2: Temporal Feature Extraction via TTCN.

The preprocessed data is first fed into the improved TCN (TTCN), which incorporates parallel dilated causal convolution layers, weight normalization, ReLU activation, and Dropout to further enhance feature extraction and generalization capabilities. The output is then passed to the attention module.

Step 3: Directional Attention via TDV Mechanism.

The output features from the TTCN are processed by the TDV attention mechanism, which adaptively adjusts the importance of each trajectory point. The attention weights remain stable during linear sailing, while dynamically emphasizing key trajectory points during sharp turns or speed variations. The attention scores are calculated based on cosine similarity between trajectory points and normalized using Softmax to ensure valid attention distributions. This mechanism enhances the model’s sensitivity to trajectory dynamics such as turning and acceleration, improving prediction accuracy.

Step 4: Sequence Modeling via BiGRU.

The BiGRU integrates forward and backward temporal dependencies. The forward GRU captures the trajectory pattern in the direction of motion, while the backward GRU uses global trajectory context to refine the reasoning process, thereby enhancing the stability and reliability of trajectory predictions.

Step 5: Trajectory Prediction via Fully Connected Layer.

The BiGRU’s output is passed through a fully connected layer for nonlinear mapping, and the predicted coordinates at the next time step are generated.

Compared with the original TCN-BiGRU model, the optimized TDV-TTCN-BiGRU model demonstrates significant advantages. The TTCN expands the receptive field using dilated convolutions while reducing computational complexity and improving parallel computing efficiency. The TDV attention mechanism enhances the model’s ability to represent directional features in trajectories, allowing for more accurate predictions across different motion states. The improved model not only achieves higher prediction accuracy but also provides a more robust solution for applications in intelligent shipping and maritime traffic management.

### Multi-level warning strategy design

During vessel navigation, environmental disturbances, maneuvering errors, or unexpected events may cause ships to deviate from their planned routes, potentially resulting in collisions with overhead transmission lines spanning waterways. Such incidents pose significant threats not only to the safety of vessels and crew but also to the stability of regional power supply systems. Therefore, the development of effective yaw warning methods is critical for the early identification and prevention of such deviations, ultimately reducing the risk of accidents and improving waterway traffic safety.

The core of this study lies in predicting ship trajectories to determine their spatiotemporal relationship with static obstacles (such as overhead power lines and bridge piers) at future time steps. When the prediction results indicate that the future trajectory of a vessel may enter the buffer zone of an obstacle or exceed the defined safety boundary, the warning mechanism is activated. In essence, trajectory prediction and yaw warning represent a proactive assessment of ship–environment interactions. Through this approach, the model can quantify potential navigation risks in advance, thereby providing reliable support for collision avoidance and waterway safety management.

**Fig 10:**
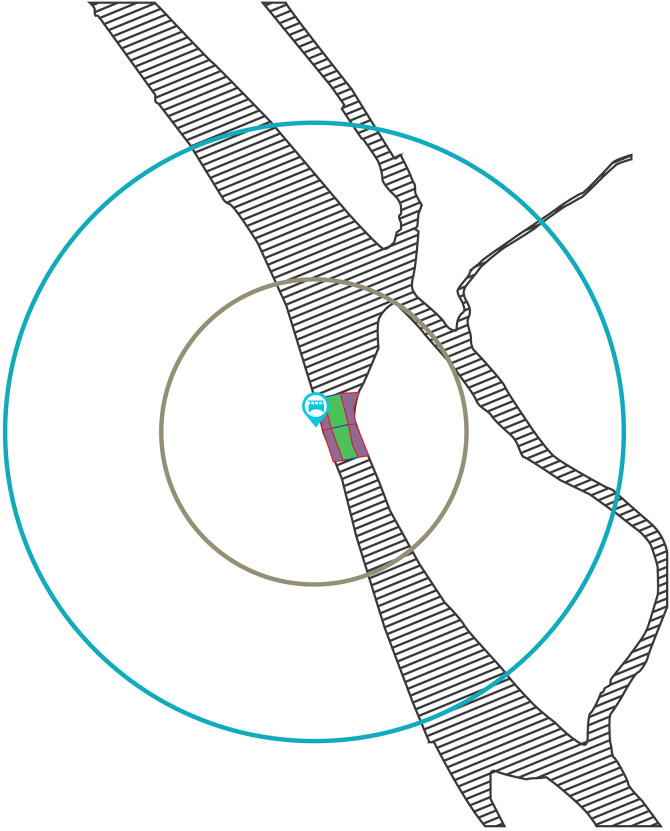
Schematic diagram of warning zone configuration.

In this study, we propose a high-efficiency yaw warning system by designing a three-tier warning mechanism based on predefined distance thresholds. The system consists of three designated zones: Warning Zone, Alarm Zone, and Emergency Alarm Zone, as illustrated in Fig 10.

The specific definitions and response strategies for each zone are as follows:

Warning Zone: This zone spans from 1.5 km to 2.0 km away from the overhead transmission line. When large and fast-moving vessels enter this region, the system issues a preliminary warning by assessing the vessel’s size and speed to indicate potential risk.

Alarm Zone: This zone covers the distance between 1.0 km and 1.5 km from the transmission line. If the vessel is oversized, moving at high speed, or shows signs of yawing, the system triggers an alarm to highlight a dangerous target.

Emergency Alarm Zone: This zone includes the area within 1.0 km of the transmission line. The presence of oversized, high-speed, or significantly deviating vessels in this region activates an emergency warning. The system recognizes the vessel as an imminent collision threat—potentially impacting the overhead conductors or support towers—and initiates emergency response procedures. These include continuous video tracking, reporting to maritime supervision authorities, and notifying relevant personnel for accident preparedness and response.

The corresponding classification of warning levels is summarized in Table 1.

**Table 1:** Warning level classification based on distance.

Distance (*l*)	Level	Response Strategy
1.5km≤l<2km	Warning Zone	Send notifications via SMS or phone call
1km≤l<1.5km	Alarm Zone	Use LED displays and warning lights for visual/electrical alerts
l<1km	Emergency Alarm Zone	Combined warning with LED displays, lights, and loudspeakers

In summary, this paper constructs a three-level yaw warning system based on distance thresholds. By dividing the monitoring region into warning, alarm, and emergency alarm zones and combining real-time vessel speed, height, and yaw status, the system dynamically evaluates the threat level posed by ships to overhead transmission lines. Corresponding warning measures are deployed based on the threat level, enabling a graded response mechanism ranging from early notification to emergency management. This approach effectively reduces the risk of vessel-contact accidents and enhances both maritime navigation and power transmission safety.

## Experiments and analysis

### Experimental environment and model parameter settings

The experiments were conducted on a Windows 11 machine using Python for data processing and model training. The deep learning models were implemented and optimized using the PyTorch framework, with auxiliary libraries such as NumPy, Pandas, and Matplotlib employed for data preprocessing and result visualization. The specific experimental environment is detailed in Table 2.

**Table 2:** Experimental environment.

Environment	Configuration
Operating System	Windows 11
CPU	AMD Ryzen 9 7945HX
GPU	NVIDIA GeForce RTX4060
RAM	16G
Programming Language	Python 3.12
IDE	PyCharm Professional 2024
Deep Learning Framework	PyTorch 2.5

The model parameter settings used in the experiments are summarized in Table 3.

**Table 3:** Model parameter settings.

Parameter	Setting
epochs	100
batch size	64
learning rate	0.001
TCN kernel size	3 × 1
Number of parallel dilated layers	3
BiGRU hidden size	128
optimizer	Adam

### Experimental data description

The experimental dataset consists of AIS trajectory data collected from a vessel with MMSI number 413773017 operating in the Yangtze River Basin. The data spans the period from November 1 to November 7, 2024, containing a total of 1,946 valid trajectory points, covering multiple round-trip voyages along the main channel of the Yangtze River. The AIS records include key attributes such as Longitude, Latitude, Speed Over Ground (SOG), Course Over Ground (COG), and Timestamps.

To ensure data quality, preprocessing procedures such as data cleaning, anomaly detection, trajectory interpolation, and normalization were applied. The dataset was divided into a training set and a test set in a 70:30 ratio, with 1,362 and 584 samples respectively, ensuring sufficient training and enabling generalization assessment on unseen trajectory sequences. A portion of the AIS data is shown in Table 4.

**Table 4:** Partial AIS data.

lon	lat	sog	cog
118.19699	31.28356	3.45	54.3
118.20796	31.28956	3.4	63.6
118.20231	31.28672	3.8	57.1
118.21382	31.29162	3.34	68.8
118.22080	31.29376	3.4	73.2
118.23113	31.29606	3.4	76.3

Here, “lat” and “lon” represent latitude and longitude, respectively, while “sog” and “cog” stand for speed over ground and course over ground, respectively.

### Evaluation metrics

#### Evaluation metrics for trajectory prediction.

To comprehensively evaluate model performance, three metrics are used: Mean Squared Error (MSE), Mean Absolute Error (MAE), and Average Displacement Error (ADE). These metrics are calculated separately for latitude and longitude to ensure a precise assessment of prediction accuracy [20]. MSE quantifies the squared difference between predicted and actual trajectory points, reflecting the overall deviation. Lower values indicate higher prediction accuracy. MAE measures the average absolute difference between predictions and ground truth, offering an intuitive sense of the average error in the predictions. The MSE and MAE are calculated as follows:


$$\begin{array}{c} MSE=\frac{\text{1}}{n}\sum \overset{n}{\underset{i=\text{1}}{\mathrm{\backslash nolimits}}}(\hat{x_{i}}-x_{i}{)}^{\text{2}} \end{array}$$ (13)

$$\begin{array}{c} MAE=\frac{\text{1}}{n}\sum \overset{n}{\underset{i=\text{1}}{\mathrm{\backslash nolimits}}}\left|\hat{x_{i}}-x_{i}\right| \end{array}$$ (14)

where n denotes the number of samples, $\hat{x_{i}}$ the predicted longitude or latitude, and $x_{i}$ the corresponding ground truth.

ADE measures the average Euclidean distance between predicted and actual trajectory points over the entire sequence [21]. It evaluates the spatial deviation of predicted trajectories.


$$\begin{array}{c} ADE=\frac{\text{1}}{n}\sum \overset{n}{\underset{i=\text{1}}{\mathrm{\backslash nolimits}}}\sqrt{{\left(\hat{x_{i}}-x_{i}\right)}^{\text{2}}+{\left(\hat{y_{i}}-y_{i}\right)}^{\text{2}}} \end{array}$$ (15)

where $\left(\hat{x_{i}},\hat{y_{i}}\right)$ and $\left(x_{i},y_{i}\right)$ are the predicted and actual longitude and latitude, respectively. Lower ADE values indicate that the predicted trajectory is closer to the actual trajectory, thus providing more reliable support for intelligent navigation.

#### Evaluation metrics for yaw warning.

To quantitatively assess the performance of the yaw warning system, this study utilizes four key evaluation metrics: Precision, Recall, F1-score, and False Alarm Rate (FAR).

(1) Precision measures the proportion of true yaw events among all the system’s yaw warnings:


$$\begin{array}{c} Precision=\frac{TP}{TP+FP} \end{array}$$ (16)

Where TP (True Positives) refers to the number of correctly predicted yaw cases, and FP (False Positives) denotes the number of false warnings where no yaw actually occurred.

(2) Recall indicates the proportion of correctly identified yaw events among all actual yaw cases:


$$\begin{array}{c} Recall=\frac{TP}{TP+FN} \end{array}$$ (17)

Where FN (False Negatives) is the number of actual yaw cases that were not detected by the system.

(3) F1-score is the harmonic mean of Precision and Recall, providing a balanced assessment of system performance:


$$\begin{array}{c} F1=\frac{2\times Precision\times Recall}{Precision+Recall} \end{array}$$ (18)

When there is a significant disparity between Precision and Recall, the F1-score offers a more comprehensive evaluation.

(4) False Alarm Rate (FAR) measures the frequency of false warnings for vessels that are not yawing:


$$\begin{array}{c} FAR=\frac{FP}{TN+FP} \end{array}$$ (19)

Where TN (True Negatives) represents the number of correctly identified non-yawing vessels. A low FAR indicates high system stability in terms of minimizing unnecessary warnings.

In summary, Precision reflects the proportion of correct alerts among all yaw warnings; a higher precision suggests the system can reduce false alarms and maintain high credibility. Recall assesses the system’s ability to detect actual yaw events; a higher recall implies better coverage of real risks. F1-score integrates both metrics and is particularly valuable when there is a trade-off between Precision and Recall. Lastly, a lower FAR signifies better robustness and fewer unnecessary disruptions. An ideal yaw warning system should aim to maintain high recall while minimizing the FAR to ensure both comprehensive detection and accurate alerts, thereby enhancing navigation safety and system reliability.

### Experimental results and analysis

#### Comparative experiments.

To evaluate the effectiveness of different models in trajectory prediction, a controlled experiment was conducted by keeping the dataset split, hyperparameters, and number of iterations constant while altering only the model structure. Five models—CNN, BiGRU, CNN-BiLSTM, CNN-BiGRU, and TCN-BiGRU—were selected for comparison. The prediction errors (MSE, MAE) for both latitude and longitude, as well as ADE, were measured. Additionally, visualizations were created to compare the predicted trajectories with the actual ones.

**Fig 11:**
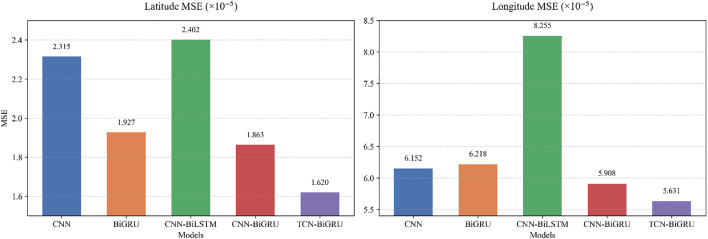
MSE of latitude and longitude for each model.

**Fig 12:**
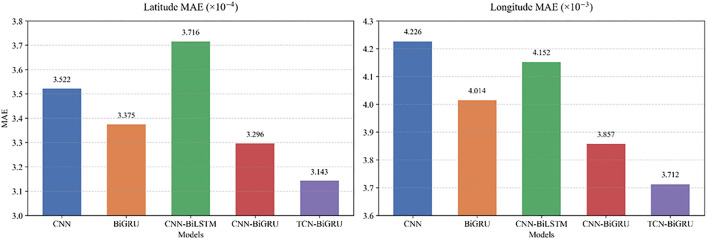
MAE of latitude and longitude for each model.

**Fig 13:**
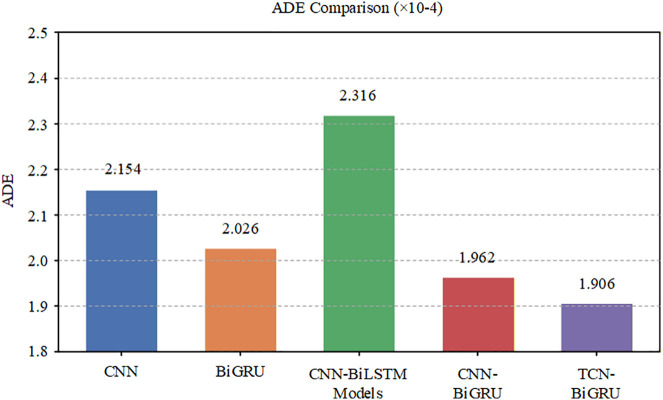
ADE of predicted trajectories.

The prediction error results using MSE are illustrated in Fig 11. When using MAE as the evaluation metric, the results are shown in Fig 12. The ADE results for each model are presented in Fig 13. The detailed error metrics for each model across the three evaluation criteria are summarized in Table 5.

**Table 5:** Performance of different models in comparative experiments.

Model	CNN	BiGRU	CNN-BiLSTM	CNN-BiGRU	TCN-BiGRU
Latitude MSE/$10^{-5}$	2.315	1.927	2.402	1.863	1.620
Longitude MSE/$10^{-5}$	6.152	6.218	8.255	5.908	5.631
Latitude MAE/$10^{-4}$	3.522	3.375	3.716	3.296	3.143
Longitude MAE/$10^{-3}$	4.226	4.014	4.152	3.857	3.712
ADE/$10^{-4}$	2.154	2.026	2.316	1.962	1.906

From Table 5, it is evident that:

In latitude prediction, the TCN-BiGRU model achieved the lowest MSE and MAE. Compared with the other four models (CNN, BiGRU, CNN-BiLSTM, and CNN-BiGRU), its MSE was reduced by 30.02%, 15.93%, 32.56%, and 13.04%, respectively, and its MAE was reduced by 10.76%, 6.87%, 15.42%, and 4.64%, respectively.

In longitude prediction, TCN-BiGRU also outperformed the others, with its MSE reduced by 8.47%, 9.44%, 31.79%, and 4.69%, and its MAE decreased by 12.16%, 7.52%, 10.60%, and 3.76%, respectively.

For ADE, TCN-BiGRU demonstrated the best overall performance, achieving the lowest displacement error with reductions of 11.51%, 5.92%, 17.70%, and 2.85% compared to the baseline models.

**Fig 14:**
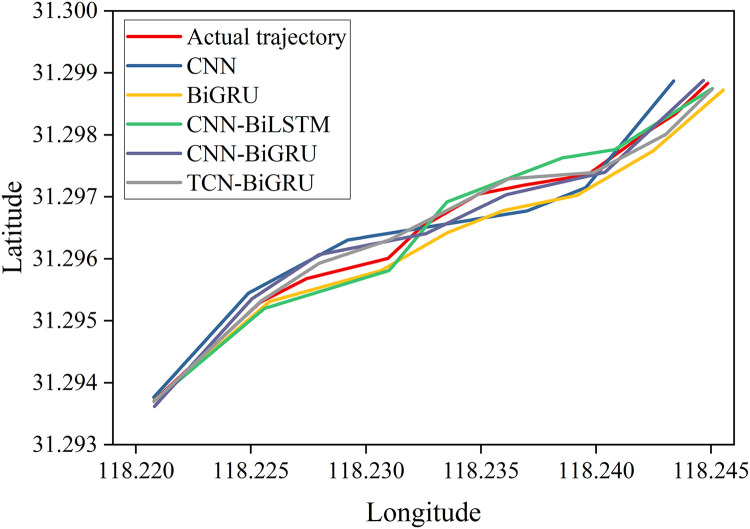
Comparison of prediction trajectories of various models in comparative experiments.

These results confirm that TCN-BiGRU exhibits superior performance across all evaluation metrics, making it more effective for ship trajectory prediction tasks. To further validate the model’s prediction capability, trajectory plots comparing the prediction results with ground truth are shown in Fig 14.

As seen in Fig 14, most models can effectively follow the general direction of the actual trajectories, particularly in straight segments at lower longitudes. However, discrepancies are noticeable in terms of accuracy. The TCN-BiGRU model shows the closest alignment with actual trajectories, especially in curves where the predicted turning angles better match the actual movement. However, in segments involving sudden maneuvers (e.g., near longitude 118.2395 and latitude 31.2979), the TCN-BiGRU model exhibits delayed longitude predictions, leading to a slight deviation of the trajectory toward the lower right. This suggests that the model’s responsiveness to abrupt directional changes remains limited, possibly due to insufficient capture of high-frequency dynamic features in the sequential data, indicating a need for further improvement.

#### Ablation experiments.

In the preceding comparative experiments, although the TCN-BiGRU model exhibited relatively high prediction accuracy across most segments, it still showed delayed responses in certain abrupt turning areas. This suggests that its adaptability to complex waterways still has room for improvement. To further enhance trajectory prediction accuracy, this study introduces a hierarchical TCN (TTCN) structure into the original TCN-BiGRU framework. The goal is to improve the model’s ability to capture long-term dependencies and reduce redundancy in high-order features. TTCN enhances temporal feature extraction by employing multiple parallel dilated causal convolution layers, enabling the model to capture multi-scale temporal patterns. This mitigates the issue of local receptive fields in traditional TCNs, thus improving overall prediction performance. This section analyzes the performance gains of the TTCN-BiGRU model over the TCN-BiGRU model through ablation experiments, using MSE, MAE, and ADE as evaluation metrics.

**Fig 15:**
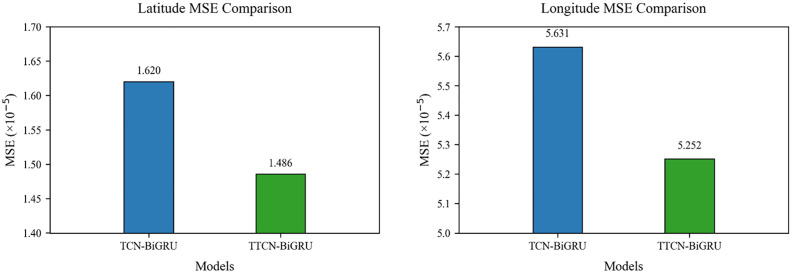
MSE of predicted latitude and longitude for each model.

**Fig 16:**
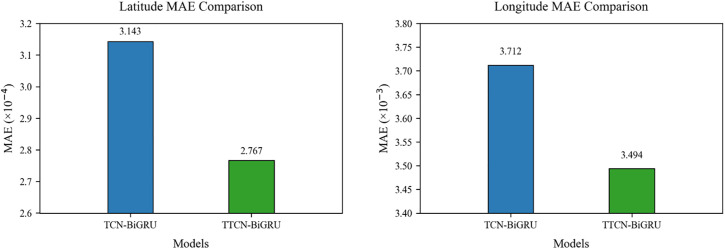
MAE of predicted latitude and longitude for each model.

The prediction errors of the two models on the dataset under different metrics are as follows. When using MSE as the evaluation metric, the results are shown in Fig 15. When using MAE as the evaluation metric, the results are shown in Fig 16.

**Fig 17:**
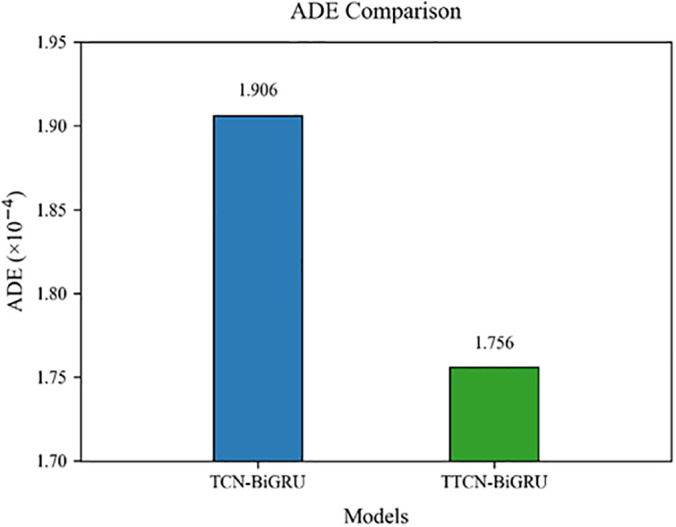
ADE of predicted trajectory positions with improved TCN.

When using ADE as the evaluation metric, the results are shown in Fig 17.

To present the error comparison more intuitively, Table 6 summarizes the specific error values of the two models under different metrics.

**Table 6:** Error performance of the improved TCN-integrated model.

Model	TCN-BiGRU	TTCN-BiGRU
Latitude MSE/$10^{-5}$	1.620	1.486
Longitude MSE/$10^{-5}$	5.631	5.252
Latitude MAE/$10^{-4}$	3.143	2.767
Longitude MSE/$10^{-3}$	3.712	3.494
ADE/$10^{-4}$	1.906	1.756

As seen from Table 6, the TTCN-BiGRU outperforms TCN-BiGRU across all metrics. Specifically, the Latitude MSE is reduced by 8.27% and the Longitude MSE by 6.73%, indicating that the hierarchical TCN structure is more effective in capturing global features of vessel trajectories. The Latitude MAE and Longitude MAE are reduced by 11.96% and 5.87%, respectively, demonstrating an improvement in modeling fine-grained local patterns. The ADE is reduced by 7.87%, further confirming that TTCN-BiGRU provides more accurate trajectory predictions.

**Fig 18:**
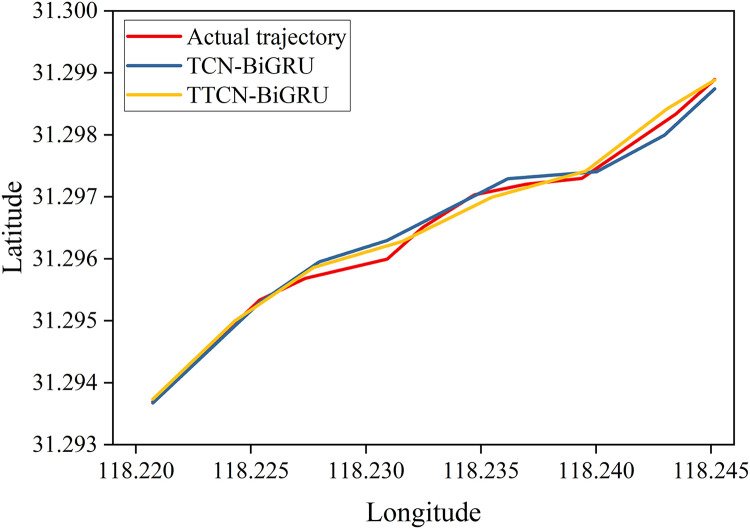
Comparison of predicted trajectories of the fusion-improved TCN model.

To further evaluate the models’ performance in real-world scenarios, a visual comparison of the predicted trajectories is presented in Fig 18.

According to the figure, both TCN-BiGRU and TTCN-BiGRU closely follow the actual trajectories, showing stable tracking performance along the main navigation segments. This indicates strong overall trend prediction capabilities. However, in critical turning regions (e.g., near longitude 118.2348), TTCN-BiGRU shows better alignment with the actual trajectory. Particularly in the segments between longitude 118.2268 to 118.2208 and 118.2370 to 118.2300, the trajectory deviation of TTCN-BiGRU is significantly reduced, verifying the enhanced adaptability of the improved model to sudden maneuvers.

Compared to TCN-BiGRU, the TTCN-BiGRU exhibits less lag in latitude and longitude predictions, though some lag still persists. This indicates that while the hierarchical TCN improves the capture of long-range temporal dependencies over traditional TCNs, its responsiveness to abrupt changes in heading and speed remains limited. To address this, a TDV attention mechanism is introduced into the TTCN-BiGRU framework to dynamically adjust the weights of ground speed and course over ground, thus improving adaptability across various navigation phases (e.g., acceleration, deceleration, turning, etc.).

The following sections compare the performance of TCN-BiGRU, TTCN-BiGRU, A-TTCN-BiGRU (with standard attention), and TDV-TTCN-BiGRU (with TDV attention). While standard attention mechanisms (A-TTCN-BiGRU) can assign varying importance to different time steps, they rely solely on global features and lack dynamic adjustment capabilities for local navigational cues such as speed and course. In contrast, the TDV attention mechanism can dynamically assign weights to trajectory-related features and adapt to various navigation conditions, thereby enhancing prediction accuracy and adaptability to sudden maneuvers.

**Fig 19:**
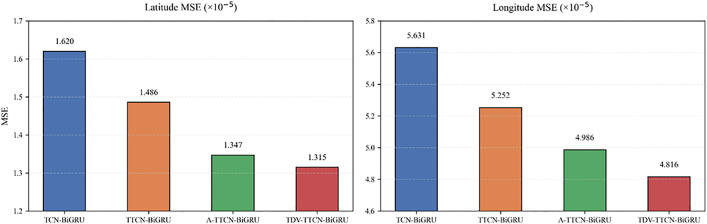
MSE of predicted latitude and longitude for each model.

**Fig 20:**
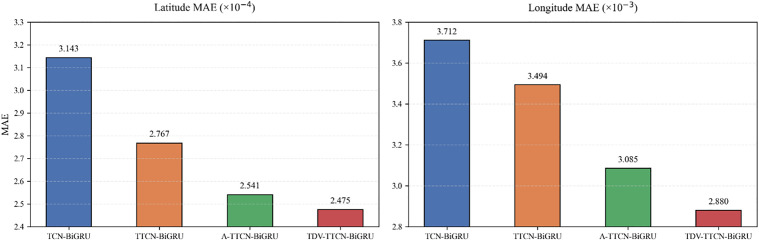
MAE of predicted latitude and longitude for each model.

**Fig 21:**
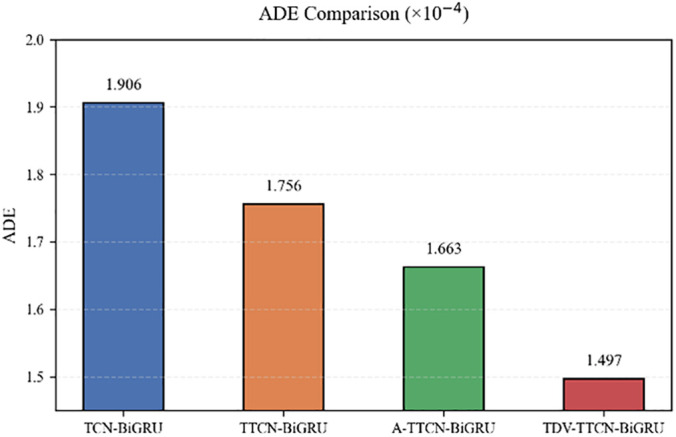
ADE of predicted trajectory positions for each model.

To analyze the impact of the TDV attention mechanism on prediction errors, we compare the models under the MSE, MAE, and ADE evaluation metrics, as shown in Figs 19, 20, and 21, respectively. Table 7 provides a numerical summary of the prediction errors for all models under each evaluation metric.

**Table 7:** Prediction errors of models with different attention mechanisms.

Model	TCN-BiGRU	TTCN-BiGRU	A-TTCN-BiGRU	TDV-TTCN-BiGRU
Latitude MSE/${\text{1}\text{0}}^{-\text{5}}$	1.620	1.486	1.347	1.315
Longitude MSE/${\text{1}\text{0}}^{-\text{5}}$	5.631	5.252	4.986	4.816
Latitude MAE/${\text{1}\text{0}}^{-\text{4}}$	3.143	2.767	2.541	2.475
Longitude MAE/${\text{1}\text{0}}^{-\text{3}}$	3.712	3.494	3.085	2.880
ADE/${\text{1}\text{0}}^{-\text{4}}$	1.906	1.756	1.663	1.497

From the table, it is evident that the TDV-TTCN-BiGRU model consistently achieves the lowest error across all metrics. For latitude prediction, the TDV-TTCN-BiGRU reduces MSE by 18.83%, 11.51%, and 2.38% compared to TCN-BiGRU, TTCN-BiGRU, and A-TTCN-BiGRU, respectively. Similarly, for MAE, the reductions are 21.25%, 10.55%, and 2.60%, confirming its superiority in latitude prediction.

For longitude prediction, the MSE of TDV-TTCN-BiGRU is reduced by 14.47%, 8.30%, and 3.41%, and MAE by 22.41%, 17.57%, and 6.65%, respectively, compared to the other three models. These results highlight the enhanced precision of the proposed model in longitude prediction.

Regarding overall trajectory accuracy (ADE), TDV-TTCN-BiGRU achieves the lowest error, with reductions of 21.46%, 14.75%, and 9.98% compared to the other models. This confirms its strong advantage in comprehensive trajectory prediction.

**Fig 22:**
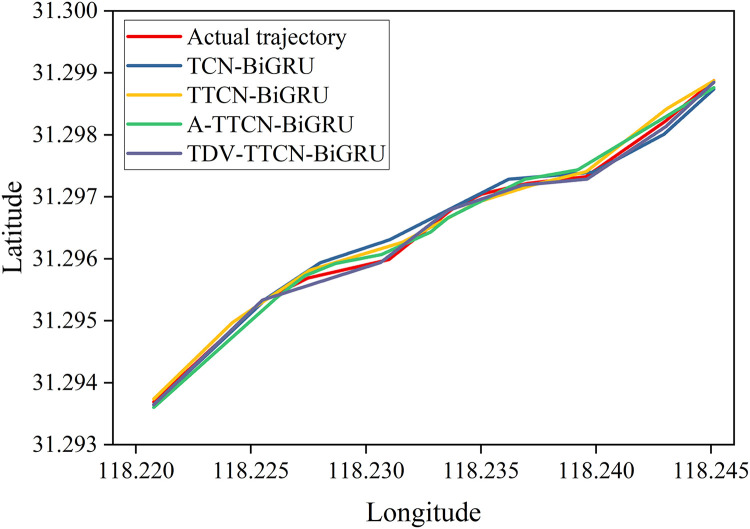
Comparison of predicted trajectories of various models in ablation experiments.

To further visualize model performance, a trajectory comparison for the four models is shown in Fig 22.

The TDV-TTCN-BiGRU model demonstrates significant advantages in both overall alignment and localized dynamic response. Compared to the baseline TCN-BiGRU and its improved versions, TDV-TTCN-BiGRU shows the highest spatial consistency with real trajectories, especially in critical turning areas (e.g., longitude 118.2340–118.2390), where it exhibits smooth transitions and accurate directional changes. At sharp turns (e.g., near longitude 118.2308), its predicted trajectory closely aligns with the actual path, resulting in superior fit.

Although A-TTCN-BiGRU enhances attention to key time steps, it fails to sufficiently focus on navigational features like speed and heading. In contrast, the TDV attention mechanism introduced in TDV-TTCN-BiGRU enables adaptive weighting of trajectory features (e.g., speed, course), allowing the model to dynamically adjust to changes in navigation behavior. This ensures timely correction of directional changes and maintains stable predictions under complex navigation scenarios.

In conclusion, the proposed TDV-TTCN-BiGRU trajectory prediction model consistently outperforms others across all evaluation metrics, fully validating the effectiveness of the hierarchical TCN structure (TTCN) and the superiority of the TDV attention mechanism. TTCN enhances the model’s ability to capture long-term dependencies through multi-layer parallel dilated causal convolutions, while TDV adaptively allocates the importance of speed and course information, significantly improving the model’s robustness and generalization, especially in response to sudden maneuvers such as turning, acceleration, and deceleration. These advantages make the model a more reliable and accurate tool for vessel trajectory prediction.

#### Yaw warning experiments.

To validate the effectiveness of the proposed multi-level yaw warning method, two experimental pilot sites were selected: the Liaoxia River section of the Dongjiang South Branch in Huizhou, Guangdong (Experiment I), and the N14–N15 river section of the 220 kV Zhanglongjia Transmission Line (Experiment II). AIS trajectory data were collected from April 1 to April 7, 2025, with 2,512 and 2,416 tracks gathered for Experiment I and II, respectively. Yaw warning responses were conducted for vessels entering and exiting the monitored areas during the observation period. The statistics of normal navigation and yaw events for the two experiments are shown in Tables 8 and 9.

**Table 8:** Statistics of normal and yaw occurrences in experiment I.

Level	Number of Occurrences
Normal Navigation	2479
Early Warning Zone	26
Alarm Zone	7
Emergency Zone	0

**Table 9:** Statistics of normal and yaw occurrences in experiment II.

Level	Number of Occurrences
Normal Navigation	2383
Early Warning Zone	27
Alarm Zone	6
Emergency Zone	0

To quantitatively evaluate the yaw warning performance of the system, this study calculated metrics such as Precision, Recall, False Alarm Rate (FAR), and F1-score for different warning levels. The results for Experiments I and II are presented in Tables 10 and 11, respectively.

**Table 10:** Evaluation results of yaw warning model in experiment I.

Warning Level	Actual Yaw Count	Correct Warnings	False Alarms	FAR	Recall	Precision	F1-score
Normal Navigation	2479	2451	19	0.77%	98.87%	99.23%	99.05%
Early Warning Zone	26	24	1	4%	92.31%	96%	94.12%
Alarm Zone	7	7	0	0%	100%	100%	100%
Emergency Zone	0	0	0	0	100%	100%	100%

**Table 11:** Evaluation results of yaw warning model in experiment II.

Warning Level	Actual Yaw Count	Correct Warnings	False Alarms	FAR	Recall	Precision	F1-score
Normal Navigation	2383	2361	16	0.67%	99.08%	99.33%	99.20%
Early Warning Zone	27	26	1	3.70%	96.30%	96.30%	96.30%
Alarm Zone	6	6	0	0%	100%	100%	100%
Emergency Zone	0	0	0	0	100%	100%	100%

The experimental results demonstrate that the proposed multi-level yaw warning model delivers high accuracy and stability across all warning levels. For both normal navigation and mild yaw, the system achieved over 99% and 96% precision, respectively, with FARs below 1%, indicating strong practical adaptability. In the alarm zone, the model achieved 100% recall and precision, showcasing its high sensitivity and responsiveness to potentially high-risk vessels.

Although no emergency-level yaw events occurred during the observation period, the model maintained zero false alarms under this highest alert tier, further validating its robustness under extreme conditions. In conclusion, the proposed system ensures high accuracy in warning issuance while effectively minimizing disturbance from false alarms. This demonstrates its excellent engineering application potential and provides critical technical support for safeguarding overhead transmission lines that cross navigable waterways.

## Conclusion

To address the challenges of insufficient long-term dependency modeling and inadequate dynamic feature weighting in inland waterway vessel trajectory prediction, this study proposes a hybrid model—TDV-TTCN-BiGRU—which integrates a TDV attention mechanism with an improved TCN-BiGRU framework. This model leverages the multi-scale temporal feature extraction capabilities of TTCN, the dynamic modeling of course and speed features via the TDV attention mechanism, and the bidirectional sequence modeling strength of BiGRU. Together, these components significantly enhance both the accuracy and stability of trajectory prediction.

The baseline TCN-BiGRU model was trained on historical AIS trajectory data using sequences of five time steps as input and benchmarked against CNN, BiGRU, CNN-BiLSTM, and CNN-BiGRU models. Experimental results demonstrate that TCN-BiGRU outperforms the others in terms of MSE, MAE, and ADE, delivering superior predictive accuracy. Building on this foundation, a refined model—TDV-TTCN-BiGRU—was developed by introducing a hierarchical TTCN structure and the TDV attention mechanism. Ablation experiments show that, compared with CNN-BiGRU, the proposed model reduces longitude and latitude MSE by 14.47% and 18.83%, MAE by 22.41% and 21.25%, and ADE by 21.46%, respectively. These improvements reflect a significantly enhanced spatial alignment between predicted and actual trajectories, demonstrating the model’s robustness and stability under complex navigation conditions.

In addition, a multi-level yaw warning method was designed to detect potential yaw events in inland waterways. Field validation was conducted in two scenarios: the Liaoxia River section of the Dongjiang South Branch and the river section under the 220 kV Zhanglongjia Transmission Line. Three spatial warning zones were defined to enable tiered responses as vessels approached overhead transmission lines. The experimental results show that the system achieved recall rates of over 92% and 100% in the warning and alarm zones, respectively, with false alarm rates under 4%, demonstrating strong performance in recognizing and responding to yaw risks in real-world scenarios.

In summary, the proposed TDV-TTCN-BiGRU model effectively improves vessel trajectory prediction accuracy, while the accompanying multi-level yaw warning system provides a reliable solution for the safety protection of overhead transmission lines across navigable waterways. The research outcomes offer theoretical and technical support for intelligent inland shipping, collision avoidance, and waterborne traffic safety management, with strong practical value and potential for broader application.

Nevertheless, the proposed method has certain limitations. The model training primarily relies on AIS data from specific river sections, resulting in insufficient diversity of data distribution. Consequently, its generalization capability under extreme hydrological conditions or complex interactive environments requires further validation. Future research may incorporate multi-source data and synthetic simulation samples to enrich the training and evaluation scenarios, thereby extending the applicability of the model to more complex waterways, multi-vessel interactions, and intelligent shipping systems, and verifying its scalability and potential for broader deployment. Moreover, given the scarcity of emergency yaw alarm events in the measured data, future work will integrate systematic evaluations based on simulation and synthetic scenarios to assess the applicability and reliability of the yaw warning mechanism under full-spectrum risk conditions.
